# Muscle Biomarkers as Molecular Signatures for Early Detection and Monitoring of Muscle Health in Aging

**DOI:** 10.3390/nu17172758

**Published:** 2025-08-26

**Authors:** Morgan LeDrew, Pauneez Sadri, Antonia Peil, Zahra Farahnak

**Affiliations:** 1 Department of Biochemistry, Memorial University, St. John’s, NL A1C 5S7, Canada; mgledrew@mun.ca; 2 Faculty of Medicine, Memorial University, St. John’s, NL A1B 3X5, Canada; psadri@mun.ca; 3 Institute of Nutritional Science, University of Potsdam, Location Rehbrücke, 14558 Nuthetal, Germany; antonia.pe@gmx.de

**Keywords:** muscle health, muscle biomarkers, myokines, sarcopenia, healthy aging

## Abstract

Maintaining muscle health is essential for preserving mobility, independence, and quality of life with age. As muscle mass and function decline, the risk of frailty, chronic disease, and disability increases. Sarcopenia, characterized by the progressive loss of muscle mass, strength, and function, is a major contributor to these adverse outcomes in older adults. Early identification and monitoring of sarcopenia are critical for timely intervention to prevent irreversible decline. Muscle biomarkers offer a promising approach for detecting muscle deterioration and guiding treatment strategies. This review explores key biomarkers—including insulin-like growth factor 1 (IGF-1), myostatin, interleukin-6 (IL-6), irisin, interleukin 15 (IL-15), and procollagen type III *N*-terminal propeptide (P3NP)—that reflect underlying processes such as muscle anabolism, inflammation, metabolism, and remodeling. Alterations in these markers are associated with muscle health status. Furthermore, hormonal status, biological sex, and nutritional factors all modulate biomarker levels, emphasizing the need for personalized assessments. Integrating biomarker analysis into clinical practice has the potential to enhance early diagnosis, inform personalized interventions, and ultimately promote healthy aging by maintaining muscle function and reducing disability risk.

## 1. Introduction

Muscle health is increasingly recognized as a cornerstone of both overall health and metabolic function [1]. It plays a vital role in physical performance and quality of life, particularly in aging populations. The process of muscle decline typically begins around the age of 40 and accelerates after the age of 60 [2]. By the age of 80, individuals can experience up to a 50% loss of muscle mass, highlighting the progressive nature of this condition [3]. This development can lead to sarcopenia, a muscle disease defined by low muscle strength, reduced muscle mass or quality, and low physical performance [4,5]. The prevalence and consequences of declining muscle health particularly affect our ever-expanding aging population [6]. Muscle decline contributes to falls, fractures, and increased mortality risk, and when coupled with excess adiposity, may progress to sarcopenic obesity, a condition characterized by the coexistence of reduced muscle mass and increased fat mass, which can exacerbate functional decline and metabolic dysregulation [7].

It is important to recognize that muscles are essential for performing daily activities such as walking, lifting, climbing stairs, and even basic tasks such as standing up from a chair [6,8]. As muscle strength diminishes, individuals may lose autonomy, becoming more reliant on others for everyday functions [9,10]. Additionally, chronic pain, impaired mobility, reduced quality of life, and declining mental health are among the most common consequences of deteriorating musculoskeletal health and sarcopenia [6]. In addition to mobility, muscles play a key role in metabolic processes, regulating energy metabolism and insulin sensitivity, thus helping prevent conditions such as type 2 diabetes, obesity, and cardiovascular disease [10,11]. Maintaining muscle health allows individuals to engage in physical activities that are essential for maintaining cardiovascular health, bone density, and overall well-being [12]. Regular physical activity, which promotes muscle health, is also linked to a lower risk of diabetes, hypertension, and obesity [13]. Preserving muscle health is thus critical for maintaining physical function and preventing chronic diseases across the lifespan [1,10,13].

Understanding muscle health requires multiple perspectives. The term “muscle health” is broad, especially when traditional measures like grip strength, muscle mass, or functional capacity are excluded [6]. Existing assessments alone may not fully capture the functional landscape of sarcopenia and other muscle-related diseases, as they may not be sensitive to subtle individual changes. Performance-based measures can be impractical in clinical settings, and invasive procedures such as muscle biopsies are often not feasible [14]. In this context, circulating biomarkers serve as a valuable, less invasive complement to traditional assessments, with all measures together providing a more comprehensive understanding to inform clinical decisions and guide therapies targeting muscle health [1]. Reliable biomarkers can also detect early signs of muscle decline—before it significantly affects daily life. As sarcopenia arises from multiple pathophysiological mechanisms, looking at a single biomarker is insufficient [15]. To provide a more comprehensive assessment, biomarkers representing key pathways, including the endocrine system, growth factors, and inflammation, have been incorporated. This approach is supported by the European Working Group on Sarcopenia in Older People (EWGSOP2), which recommends investigating panels of biomarkers spanning multiple relevant biological systems [4]. While the ongoing challenge related to the standardization of biomarker measurement—including assay variability, time-of-day effects, and population heterogeneity—is acknowledged, biomarkers are included based on their biological relevance and use in recent literature. In particular, several molecules have been identified as critical regulators of muscle growth, repair, and metabolism. These include insulin-like growth factor (IGF-1), myostatin, interleukin-6 (IL-6), irisin, interleukin-15 (IL-15), and procollagen type III *N*-terminal propeptide (P3NP). For instance, while IGF-1 promotes muscle proliferation and differentiation and inhibits protein breakdown, myostatin inhibits muscle proliferation and differentiation. IL-6 can act as both a pro- and anti-inflammatory cytokine and is involved in muscle repair and regeneration. Irisin promotes adipose tissue browning and supports muscle metabolism, and IL-15 contributes to muscle proliferation and regeneration, enhancing oxidative capacity. P3NP reflects collagen turnover and muscle remodeling. Monitoring these biomarkers can help us understand muscle function and track the development of sarcopenia, ultimately supporting earlier diagnosis and more targeted interventions.

### Objectives

This review aims to evaluate the role of circulating biomarkers in maintaining muscle health and preventing sarcopenia. Sarcopenic obesity is also an important consideration; the combination of sarcopenia and excess adipose tissue, which further compromises muscle function, exacerbates metabolic dysregulation, and increases the risk of poor health outcomes. A clear understanding of muscle physiology and composition, including the balance between muscle protein synthesis and degradation, the role of connective tissue remodeling, and the interaction between muscle and adipose tissue, is critical for developing effective prevention and treatment approaches.

The primary objective of this review is to synthesize current evidence on several key biomarkers involved in muscle health, focusing on their potential roles in preventing age-related muscle decline. Specifically, we review IGF-1, myostatin, IL-6, irisin, IL-15, and P3NP, summarizing their biological and metabolic roles, clinical relevance, and potential applications in early detection and treatment monitoring. By reviewing these biomarkers in the context of both sarcopenia and sarcopenic obesity, this review aims to provide a foundation for targeted, personalized strategies that integrate biomarker monitoring with routine physical activity and optimal nutrition to help maintain muscle health and improve quality of life in older adults.

## 2. Sarcopenia and Sarcopenic Obesity

Sarcopenia, characterized by the progressive loss of skeletal muscle mass, strength, and function, is a prevalent condition among aging populations and poses a significant challenge to overall muscle health [4,5]. This age-related decline in muscle results from a complex interplay of various factors, including hormonal changes, reduced physical activity, and impaired protein metabolism, all of which negatively impact quality of life and increase mortality [16]. As muscle mass diminishes, strength and function also decline, increasing vulnerability to falls, fractures, and frailty [8,17]. Sarcopenia is further linked to metabolic disturbances—such as insulin resistance, dyslipidemia, and obesity—which increase the risk of chronic diseases such as type 2 diabetes and cardiovascular conditions [11]. The resulting metabolic dysfunction creates a vicious cycle, where muscle atrophy further disrupts metabolic processes, accelerating muscle deterioration [10].

When sarcopenia coexists with obesity, it manifests as sarcopenic obesity, a condition marked by the simultaneous presence of muscle loss and excess fat accumulation, and is particularly concerning [18]. It complicates health management by combining the physical limitations of sarcopenia with the metabolic challenges of obesity. The pathophysiology of these conditions involves complex interactions between muscle and fat cells. Muscle releases myokines and fat tissue releases adipokines, both of which can interfere with the balance of muscle and fat metabolism [16]. This disruption is a key driver of disease progression and highlights the need for treatment strategies that target both muscle and metabolic health.

**Figure 1:**
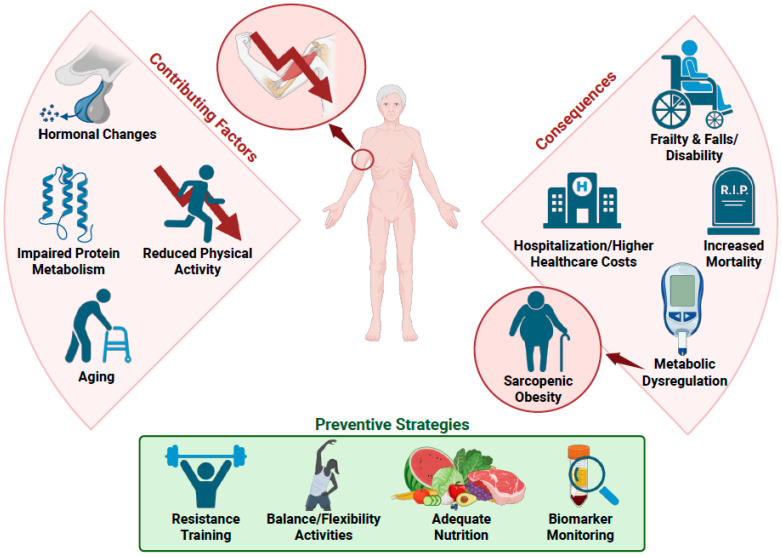
Sarcopenia: causes, consequences, and preventive strategies. Summary of the key factors contributing to sarcopenia, its associated health outcomes, and evidence-based strategies for prevention and management. Created with BioRender.com.

Together, sarcopenia and sarcopenic obesity place a significant burden on healthcare systems, especially in aging populations. As muscle declines with age, the risk of frailty, disability, hospitalization, and long-term care increases [10,17,19]. These consequences translate into higher healthcare costs due to increased demand for medical interventions, rehabilitation, and institutional care [20]. Early prevention and management strategies of sarcopenia could help reduce this burden while improving quality of life [9]. It is well established that preventing sarcopenia requires a multifaceted approach, combining regular physical activity with adequate nutrition. Resistance training, such as weightlifting and body-weight exercises, is essential for boosting strength and muscle mass [10,21,22]. Activities such as yoga also help by improving balance and flexibility, reducing the risk of falls [22]. Dietary strategies are equally important. A recent study showed that a short-term low-calorie diet with adequate protein intake, combined with whey protein, leucine supplementation, and an optimal combination of micronutrients, such as vitamin D, promoted muscle mass and strength in post-menopausal women with sarcopenic obesity [23]. Adequate protein supports muscle repair and growth, and nutrients such as vitamin D, calcium, and omega-3 fatty acids contribute to both muscle and bone health [21,22]. A diet with low inflammatory potential has been shown to be associated with greater muscle strength and mass in community-dwelling older adults of both sexes [24]. Importantly, community-based programs that provide access to exercise facilities, nutritional education, and health screenings can promote healthy lifestyle habits that support muscle health throughout aging [22]. Integrating these strategies, along with efforts to identify reliable biomarkers, will be crucial in preserving muscle. Biomarkers can help detect muscle decline early, assess intervention effectiveness, and support personalized strategies to enhance physical function and quality of life (Figure 1).

## 3. Muscle Physiology and Composition

As the body’s most abundant tissue, skeletal muscle plays a key role in movement, posture, and energy metabolism; therefore, muscle health is closely linked to systemic processes, including metabolic regulation and inflammation [10].

Muscle health is defined by three core elements: mass, strength, and function. Muscle mass refers to the total amount of skeletal muscle tissue, which is essential for physical strength and mobility [10,25]. As people age, muscle mass can decrease due to mitochondrial dysfunction and oxidative stress, contributing to atrophy and reduced muscle quality [26,27]. Muscle strength, the ability of muscles to exert force, determines an individual’s capacity to perform physical tasks. Loss of strength can impair the ability to engage in daily activities such as standing up or climbing stairs [5,28]. Lastly, muscle function encompasses the efficiency and coordination with which muscles perform these activities and is crucial for maintaining independence and quality of life [10,29]. Importantly, muscle tissue does not function in isolation. It interacts closely with adipose tissue, which plays a pivotal role in energy storage, endocrine signaling, and inflammation regulation [30]. Adipose tissue secretes a range of bioactive molecules known as adipokines, which can influence muscle metabolism. For instance, pro-inflammatory adipokines such as leptin and resistin are associated with muscle wasting, whereas beneficial adipokines such as adiponectin improve insulin sensitivity and enhance muscle performance [31,32,33]. The relationship between muscle and adipose tissue is bidirectional: muscle also influences adipose tissue function through the secretion of myokines. These myokines regulate fat metabolism and may help combat obesity-related disorders [34]. However, with aging, this balance shifts: visceral fat tends to increase while muscle mass declines, contributing to impaired mobility [35]. This interplay underscores the need to maintain a healthy balance between muscle and adipose tissue to support overall metabolic health. In the context of aging and chronic disease, the changes in fat distribution and accumulation can further exacerbate muscle decline, making the crosstalk between muscle and fat tissues a central factor in age-related frailty.

**Figure 2:**
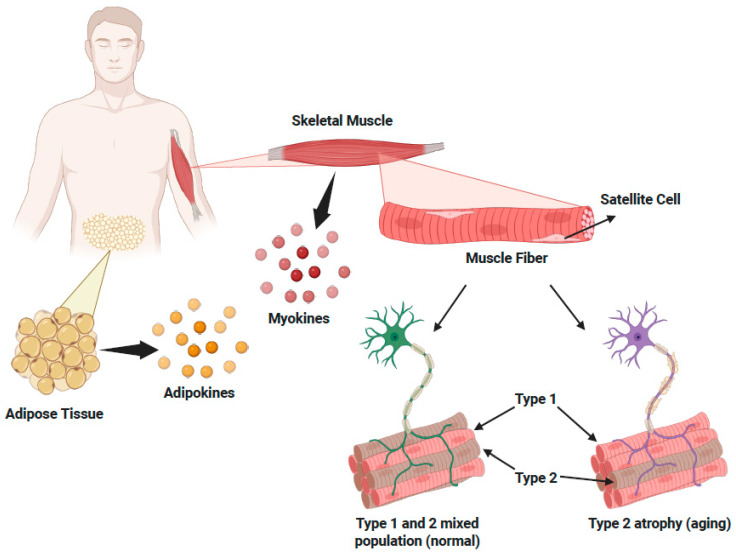
Muscle fiber composition and its alteration with aging. Crosstalk between adipose and skeletal muscle tissue occurs through the release of adipokines and myokines, which influence metabolic health and muscle maintenance. Within skeletal muscle, satellite cells support muscle regeneration and are essential for preserving muscle mass with age. Aging leads to shifts in muscle fiber composition, including type II fiber atrophy and a relative increase in type I fibers, contributing to reduced muscle strength and function. These changes are key features of sarcopenia and are further exacerbated by increased adiposity (Adapted from [18,39]). Created with BioRender.com.

Evidence suggests that muscle composition is a critical factor in assessing age-related muscle decline, reflecting the structural and functional changes that occur with aging. These changes go beyond simple reductions in muscle mass and include alterations in muscle fiber type, contractile properties, and metabolic capacity. Accordingly, muscle fibers are classified based on their contractile speed and metabolic activity. Type I fibers are oxidative and endurance-oriented, while type II fibers are adapted for rapid, powerful movements [36]. The balance between muscle protein synthesis and degradation is also essential for maintaining mass, as disruptions in this balance lead to sarcopenia [18]. Additional contributors to muscle health include fiber size, shape, arrangement, metabolic enzyme activity, and protein content—all of which can change with age. Thus, monitoring changes in muscle strength, mass, and function over time is critical for early detection of decline. Individuals with poor muscle quality often exhibit reduced specific force production and slower contraction velocities, particularly in type II fibers [37]. Analyzing changes in muscle fiber properties is not only useful for quantifying muscle loss but also for designing targeted interventions to slow or reverse decline. In adults, muscle satellite cells have the unique capacity to repair and regenerate muscle fibers [38]. These cells are usually inactive but become activated in response to muscle injury [38]. As we age, the regenerative capacity of these satellite cells declines, contributing to sarcopenia [38]. Strategies aimed at preserving satellite cell function—or introducing rejuvenated progenitor cells—may offer therapeutic options to prevent or reverse age-related muscle loss [38] (Figure 2).

Assessing muscle health and composition in middle-aged adults offers a window of opportunity for early intervention. Regular physical activity can attenuate the adverse effects of aging on muscle composition and metabolic efficiency [40]. Developing comprehensive, objective tools to assess muscle quality could enhance personalized interventions and promote healthy aging at the population level.

## 4. Myokines as Biomarkers of Muscle Health

Myokines—proteins secreted by muscle cells—are emerging biomarkers of muscle health due to their role as signaling molecules released by active muscle cells [9]. Key examples (IGF-1, myostatin, IL-6, irisin, IL-15, and P3NP) influence metabolism, energy expenditure, lipid metabolism, and insulin sensitivity, underscoring the central role of muscle in systemic homeostasis [9,18]. They also regulate muscle differentiation, regeneration, mitochondrial function, and inflammation [41]. Human and animal studies show circulating myokine levels respond to exercise, nutrition, and disease states, often correlating with changes in muscle mass or function. Thus, myokines offer insight into frailty mechanisms and support the development of interventions to preserve muscle function and quality of life in aging.

### 4.1. Insulin-like Growth Factor-1 (IGF-1)

#### 4.1.1. Metabolic Role of IGF-1

Among myokines, IGF-1 is particularly important because of its dual role in promoting muscle growth and inhibiting degradation. IGF-1 is primarily produced in the liver in response to growth hormone (GH), but it is also synthesized locally within skeletal muscle [42]. In muscle, IGF-1 acts through autocrine and paracrine mechanisms to promote the proliferation, differentiation, and survival of muscle cells [43]. It also enhances protein synthesis by activating key intracellular signaling pathways, such as the phosphoinositide 3-kinase (PI3K)/Akt pathway, which in turn stimulates the mammalian target of rapamycin (mTOR) pathway—a critical regulator of muscle protein turnover [1,44]. In addition, IGF-1 activates muscle satellite cells (MSCs), which are essential for the repair and regeneration of muscle following injury or stress; therefore, IGF-1 is especially significant in injuries and catabolic conditions, as its synthesis increases in MSCs following muscle injury while also stimulating MSC proliferation [1,45].

Beyond its anabolic effects, IGF-1 also plays a role in mitigating muscle protein breakdown through inhibition of catabolic pathways. For example, IGF-1 suppresses the activity of the ubiquitin–proteasome system, a major pathway for muscle protein degradation [46]. This dual effect—stimulating growth while inhibiting degradation—makes IGF-1 a valuable marker when evaluating interventions aimed at preserving or enhancing muscle health, including exercise programs, nutritional strategies, and pharmacological therapies. However, the clinical utility of IGF-1 as a biomarker remains under investigation, especially in the context of sarcopenia [18].

#### 4.1.2. IGF-1, Muscle Growth, and Exercise

Lower IGF-1 has been correlated with reduced muscle mass, poor physical performance, and increased muscle weakness—hallmark features of sarcopenia [10]. IGF-1 deficiency may also impair the capacity of muscle tissue, delaying recovery and diminishing overall functional ability [47]. This has particular relevance in aging populations, where natural declines in GH levels contribute to reduced circulating IGF-1 production and diminished regenerative function. In a human study of 200 community-dwelling Asian adults aged 50–99 years, dual-energy X-ray absorptiometry (DXA) was used to assess lean mass, and serum IGF-1 was also measured [48]. Lower IGF-1 levels were associated with frailty and reduced muscle mass in women (effect size: small; *p* < 0.05), suggesting potential sex-specific effects, though the magnitude of association was modest and may have limited clinical significance [48]. Conversely, elevated IGF-1 levels have been associated with increased muscle mass and strength and are known to promote hypertrophy following resistance training [49]. However, in a human randomized controlled trial involving 33 untrained women aged 33.6 ± 9.2 years, twelve weeks of combined resistance and interval training significantly improved muscle strength and lean mass but did not change IGF-1 concentrations (*p* > 0.05) [50].

In another human randomized controlled trial of 62 sedentary Brazilian men aged 65–75 years, a 24-week intervention study compared moderate-intensity and high-intensity resistance training to a non-training control group. Both training groups showed significantly increased serum IGF-1 levels versus controls (moderate-intensity: *p* = 0.02; high-intensity: *p* < 0.001), with a large effect size in the high-intensity group (Cohen’s d ≈ 0.8). Clinically, these changes were accompanied by significant gains in strength and lean mass, indicating both statistical and functional significance [51]. The findings suggest that resistance training, especially at higher intensities, can effectively enhance IGF-1 levels in older sedentary men. Similarly, a recent randomized trial involving 61 older adults (70.6 ± 4.7 years; 47% men) undergoing an 8-week vibration and home-based resistance exercise program also showed improvements in muscle outcomes only in men. Participants were assigned to exercise alone, exercise plus high-protein, or exercise plus high-protein with omega-3. High-protein with exercise improved leg strength and chair rise time (*p* < 0.05), while adding omega-3 increased IGF-1 and reduced IL-6 in men only (*p* < 0.05), suggesting that combined high-protein and omega-3 supplementation with exercise enhances muscle power and reduces inflammation in older men [52].

In summary, IGF-1 is a multifaceted and vital biomarker for assessing muscle health. Monitoring its levels can offer insight into the anabolic and metabolic status of skeletal muscle and help predict age-related muscle decline. It can also serve as a useful tool for tracking the effectiveness of muscle-preserving interventions, including exercise and nutrition. However, further research is needed to better understand the complex mechanisms regulating IGF-1 in muscle health and disease [10].

### 4.2. Myostatin

#### 4.2.1. Metabolic Role of Myostatin

Myostatin belongs to the transforming growth factor-beta (TGF-β) superfamily. It inhibits muscle cell proliferation and differentiation and is considered a well-established negative regulator of muscle growth [10]. Myostatin exists as an inactive precursor that becomes biologically active when its *C*-terminal domain dimerizes through proteolytic cleavage, allowing secretion into the bloodstream [53]. While the exact mechanism of action of its activation remains unclear, evidence suggests that multiple ligands interact with myostatin to fine-tune the control of muscle growth [53]. For instance, myostatin exerts its inhibitory effects by binding to its receptor, activin receptor type IIB (ActRIIB), and then the mature myostatin peptide activates downstream signaling pathways such as the suppressor of mothers against decapentaplegic (SMAD). The mature myostatin peptide activates SMAD proteins, which mediate intracellular signaling, enabling myostatin to translocate into the nucleus and activate transcription of target genes [53]. The SMAD signaling ultimately suppresses the Akt/mTOR pathway, which is essential for promoting muscle protein synthesis and preventing muscle atrophy [16]. The mature myostatin peptide is also implicated in MAPK cascades, both SMAD-dependent and independent [53].

#### 4.2.2. Myostatin and Muscle Wasting

Animal research has helped define myostatin’s physiological and pathological roles. In a controlled animal study using myostatin-knockout mice, both muscle fiber hyperplasia and hypertrophy were observed, with these effects persisting into adulthood, highlighting myostatin’s involvement in both early muscle development and long-term growth regulation [53]. In another animal study, adult male Sprague-Dawley rats were given an intramuscular electro-transfer of a myostatin expression plasmid in the tibialis anterior muscle, with the contralateral leg receiving an empty vector as a control. Muscle samples were collected 7 and 14 days post-intervention, and the myostatin-overexpressing muscle showed significant atrophy, reduced fiber size, and decreased protein content without a change in fiber number [54]. The muscle atrophy was accompanied by downregulation of structural and myogenic genes, emphasizing myostatin’s central role in inducing muscle loss under both physiological and pathological conditions.

Expanding observations from preclinical models, human studies show that in healthy individuals, myostatin levels remain low to moderate, ensuring that muscle growth is regulated appropriately [18]. However, studies show that myostatin levels increase with age and are associated with reduced muscle mass and functional performance—hallmark features of sarcopenia [10]. Moreover, myostatin is believed to play a role in the failure of muscle regeneration in older individuals by inhibiting satellite cell activation and preventing effective tissue repair after injury [55]. For instance, in a large human observational study involving 463 community-dwelling adults aged 53–92 years in Taiwan, serum myostatin, muscle mass, strength, and function were measured according to Asian Working Group for Sarcopenia (AWGS) criteria [56]. Lower serum myostatin levels were significantly associated with reduced muscle mass in men (*p* < 0.05), but not in women, suggesting a sex-specific relationship. Multivariate analysis confirmed myostatin as an independent predictor of muscle decline in men, although the effect size was modest [56]. Myostatin’s role as a biomarker also extends to monitoring the outcomes of therapeutic interventions. Exercise training and certain nutritional interventions have been shown to lower myostatin expression, supporting muscle growth and recovery [57,58]. Conversely, persistently elevated myostatin levels in individuals with muscle-related disorders may signal ongoing degeneration or poor treatment response. Extending this evidence, a recent case–control study of 40 patients with distal radius fractures (20 sarcopenic, mean age 63.4 ± 8.1 years; 20 age- and sex-matched non-sarcopenic controls, 62.1 ± 7.9 years) showed that sarcopenic patients exhibited significantly lower vitamin D receptor gene and protein expression in skeletal muscle (qRT-PCR *p* < 0.01; Western blot 2.1-fold higher in controls, *p* < 0.01) and higher myostatin expression (qRT-PCR and Western blot, *p* < 0.05). These findings suggest that impaired muscle regenerative capacity, mediated in part by dysregulated myostatin and vitamin D signaling, is closely linked to sarcopenia [59].

In summary, myostatin is recognized not only as a potential biomarker for muscle status but also as a promising therapeutic target for managing muscle degenerative diseases [10,18]. Thus, understanding myostatin’s regulatory functions and its interplay with other anabolic and catabolic signals remains essential for developing better diagnostic tools and targeted therapies aimed at preserving muscle.

### 4.3. Interleukin-6 (IL-6)

#### 4.3.1. Metabolic Role of IL-6

IL-6 is a cytokine with a key role in muscle health, particularly in the regulation of inflammation and muscle metabolism. IL-6 is produced by skeletal muscle cells in response to exercise, injury, and other stimuli, and exhibits both pro-inflammatory and anti-inflammatory effects, depending on the physiological context [60]. In healthy individuals, IL-6 levels are typically low, but they rise in response to physical activity or muscle damage. In these settings, IL-6 helps regulate muscle repair and regeneration by promoting satellite cell activation and facilitating tissue recovery [61]. However, chronic or excessive IL-6 production contributes to muscle degradation, especially under conditions of prolonged systemic inflammation. Elevated IL-6 levels have been linked to muscle wasting disorders, such as sarcopenia and cachexia, in which sustained inflammation accelerates muscle atrophy and impairs regenerative processes [62]. Mechanistically, IL-6 influences muscle catabolism through the modulation of signaling pathways involved in muscle catabolism, including the activation of nuclear factor-kappa B (NF-κB) and the Janus kinase/signal transducer and activator of transcription (JAK/STAT) pathways. These pathways promote the expression of genes involved in protein degradation [63].

#### 4.3.2. IL-6 Dual Role

IL-6 concentrations may serve as a biomarker of muscle inflammation and overall muscle function, making it a useful tool for assessing disease severity in muscle-wasting conditions [18]. Interestingly, IL-6 also plays a dual role in muscle metabolism, as it may promote muscle hypertrophy under certain conditions, such as acute exercise, underscoring its complex involvement in muscle regulation [10]. However, in aging populations, chronically elevated IL-6 levels have been strongly associated with the development and progression of sarcopenia, as persistent inflammation disrupts muscle homeostasis, leading to both muscle mass loss and functional decline [64]. In line with these findings, in a cross-sectional human study involving 70 community-dwelling women aged 65–70 years, elevated IL-6 and C-reactive protein (CRP) levels were independently associated with lower skeletal muscle mass (*p* < 0.05), even after adjusting for waist circumference, protein intake, physical activity, and muscle-strengthening activity [65]. No significant association was found in men, suggesting a potential sex-specific interaction between inflammatory markers and muscle mass.

Endocrine factors, particularly sex hormones, may also contribute to changes in muscle health and inflammation during aging. Testosterone is an anabolic hormone that promotes muscle protein synthesis, while estrogen has been shown to reduce muscle inflammation following injury and to facilitate muscle repair responses [66,67]. Therefore, the postmenopausal reduction in estrogen is thought to have a greater negative impact on muscle health in women, whereas the age-related decline in testosterone among men appears to have a less pronounced effect [66,68]. For instance, in a randomized human trial of 108 men aged approximately 66 years with low-normal testosterone, participants received either placebo or testosterone gel at two target ranges (400–550 ng/dL or 600–1000 ng/dL) for 12 months, with or without progressive resistance training [69]. Testosterone therapy improved body composition (reduced fat mass and increased fat-free mass; *p* < 0.05) but did not significantly enhance functional performance [69]. Similarly, in another human randomized controlled trial of 274 frail older men (≥65 years) transdermal testosterone (50 mg/day) for six months significantly increased lean mass (mean gain 1.5 kg; *p* < 0.001), reduced fat mass (*p* < 0.001), and improved lower limb strength (*p* < 0.05), with modest improvements in physical function [70]. These findings suggest testosterone therapy may help counteract age-related declines in muscle strength and body composition, particularly in frail older men [70]. Furthermore, the influence of sex hormones on female muscle health has been assessed in a recent cross-sectional study of 319 females (aged 24–89 years) and a longitudinal 4–6 years of follow-up of 83 females over 50 years. Higher estradiol (E2) and free estradiol index (FEI) were associated with greater lean mass and lower body fat (β = 0.20–0.28 and −0.21–−0.30, *p* < 0.05). Bioavailable testosterone (BioT) was linked to higher lean mass and body fat, while total testosterone (TT) negatively correlated with body fat. Longitudinal declines in E2 and FEI predicted decreases in lean mass and handgrip strength, and declines in TT and BioT were associated with increased body fat (β = 0.21–0.41 and −0.25, *p* < 0.05). Their findings suggest that sex hormone levels are closely associated with muscle mass, composition, and function in females, highlighting their role in muscle ageing [71]. Overall, these studies highlight the role that sex hormones play in maintaining muscle health.

In summary, IL-6 is an important biomarker for muscle health due to its regulatory role in inflammation, repair, and regeneration. While it supports muscle recovery following exercise or injury, chronically elevated levels are associated with muscle wasting and functional decline, particularly in aging and inflammatory muscle diseases. Monitoring IL-6 levels can provide insights into muscle health and serve as a valuable tool for evaluating the effectiveness of interventions aimed at preserving muscle health [9,10,18].

### 4.4. Irisin

#### 4.4.1. Metabolic Role of Irisin

Irisin is produced primarily during physical activity when the hormone fibroblast growth factor 21 (FGF21) is released and activates peroxisome proliferator-activated receptor gamma coactivator 1-alpha (PGC-1α) in skeletal muscle cells [72]. This activation induces irisin gene expression, after which irisin is secreted into circulation, where it can exert systemic effects, including the promotion of adipose tissue browning and the stimulation of mitochondrial biogenesis in muscle cells [73]. Irisin has been linked to muscle growth and repair by stimulating mitochondrial activity and enhancing muscle metabolism, especially in response to physical activity [10]. Irisin also exhibits protective effects against muscle atrophy and the decline in muscle function associated with aging and various muscle-wasting conditions [18].

#### 4.4.2. Irisin and Muscle Function

Elevated irisin concentrations are typically observed following acute bouts of exercise. Several studies suggest that higher circulating irisin levels correlate with greater muscle strength, endurance, and enhanced recovery [74,75]. In aging, where muscle loss and frailty are prevalent, irisin may serve as a marker of muscle health [10]. Moreover, irisin’s ability to promote muscle regeneration and counteract oxidative stress further underscores its potential as a biomarker for assessing muscle function, particularly in individuals at risk for muscle degeneration [18]. In a randomized human intervention study involving 17 older men (mean age 62), participants were assigned to a control group (*n* = 7) or a leg and core strength training group (*n* = 10). The training program, performed twice weekly for 55 min over 12 weeks, significantly increased serum irisin levels in the exercise group (*p* < 0.05), while no changes occurred in the control group. Increases in irisin were negatively correlated with whole-body fat percentage (r = −0.71, *p* < 0.05), suggesting that higher irisin may also help regulate adiposity in older adults [76].

In summary, irisin’s role in both muscle repair and metabolic regulation provides valuable insight into muscle function. Its circulating levels are influenced by physical activity and can reflect changes in muscle strength, making it a promising biomarker for diagnosing and monitoring the effects of exercise and other interventions targeting muscle preservation [9,10,18].

### 4.5. Interleukin-15 (IL-15)

#### 4.5.1. Metabolic Role of IL-15

IL-15 is a pro-inflammatory myokine and is predominantly produced by skeletal muscle in response to exercise. It plays a key role in stimulating muscle cell proliferation, enhancing muscle regeneration, and improving the oxidative capacity of muscle fibers [10]. IL-15 regulates key metabolic processes, such as mitochondrial biogenesis, protein synthesis, and muscle cell differentiation [18,77]. Elevated levels of IL-15 have been associated with enhanced muscle strength and an increase in lean body mass [78]. Studies have shown that individuals undergoing physical training or rehabilitation exhibit higher IL-15 levels, which correlate with better functional outcomes [18]. As such, IL-15 may serve as a useful biomarker for monitoring muscle function, assessing recovery from exercise, and assessing progression in muscle-related diseases—especially in aging populations or individuals with chronic conditions leading to muscle wasting [10,18].

#### 4.5.2. IL-15 and Body Composition

IL-15 also functions as a circulating myokine that helps reduce fat storage in the body by inhibiting adipose tissue deposition [79]. In an animal study, male and female transgenic mice engineered to overexpress IL-15 in skeletal muscle were weaned at 3 weeks, placed on a low-fat/low-energy diet (10% fat, 3.8 kcal/g) for 5 weeks, and then randomized to either remain on the same diet or switch to a high-fat/high-energy diet (60% fat, 5.2 kcal/g) for 21 weeks. IL-15 overexpression protected against diet-induced obesity by reducing fat accumulation, particularly in white adipose tissue, and promoting lean body mass retention [79]. In male mice, IL-15 suppressed fat gain even under a high-fat diet, while in females it promoted lean mass gain regardless of diet type, suggesting possible sex-specific effects on body composition regulation. These results highlight IL-15’s dual role in muscle anabolism and fat metabolism and its potential importance in preserving a favorable body composition, particularly during aging or periods of reduced physical activity [79]. In the context of aging, a human observational study involving 123 community-dwelling older adults showed that plasma IL-15 concentrations were significantly lower in individuals with sarcopenia compared to controls (*p* < 0.001). Multivariate analysis showed that lower IL-15 levels were independently associated with sarcopenia, along with age and body mass index, suggesting that reduced IL-15 may be an indicator of age-related muscle loss [80].

In summary, IL-15 is involved in muscle maintenance, fat metabolism, and regulation. Its roles in mitochondrial function, protein synthesis, and muscle repair support its use as a biomarker of muscle health [10,18]. Lower IL-15 levels have been linked to worse muscle health outcomes, particularly in older adults [80]. These findings suggest that IL-15 may be a valuable tool for monitoring body composition across the lifespan.

### 4.6. Propeptide of Type III Procollagen (P3NP)

#### 4.6.1. Metabolic Role of P3NP

Collagen is a crucial component of the muscle’s structural framework, supporting the growth and remodeling of myoblasts during muscle repair [81]. During collagen synthesis, cleavage of the *N*- and *C*-terminal yields the P3NP, which is released into circulation in direct proportion to collagen production [82]. Since P3NP can be easily measured in the blood and reflects muscle remodeling, it serves as a potential biomarker of anabolic activity and muscle loss [83].

#### 4.6.2. P3NP and Muscle Gain

In a randomized human trial of 23 healthy adults aged 61–85 years, participants were assigned to either a supervised resistance training group or a non-exercising control group [83]. The intervention involved individualized, twice-weekly, full-body strength training sessions. While the exercise group showed significant improvements in muscle strength and quality (*p* < 0.05), changes in circulating P3NP levels were modest and not statistically significant—a 7.9% increase in the exercise group versus 1.9% in controls (*p* > 0.05). However, baseline P3NP levels were positively correlated with gains in lean body mass (r = 0.42, *p* = 0.045), suggesting that individuals with higher initial P3NP may be more responsive to training in terms of lean mass gain. No significant associations were found between P3NP and changes in muscle strength or quality [83]. In a cross-sectional human study of Taiwanese adults aged ≥65 years, sarcopenia was assessed using the AWGS (2019) criteria—incorporating hand grip strength, skeletal muscle mass (via bioelectrical impedance analysis), and gait speed. Higher P3NP levels were significantly associated with greater skeletal muscle index (SMI) in men (*p* < 0.05), but this association disappeared after full adjustment for confounders. When evaluated as a diagnostic tool for sarcopenia, P3NP had only modest discriminatory ability (AUROC = 0.578), with optimal cut-off points of 0.045 in men and 0.035 in women [84]. Thus, while P3NP shows some association with skeletal muscle mass, additional biomarkers or combined approaches may be necessary for accurate sarcopenia detection in older adults.

Previous research has also demonstrated that P3NP increases in response to testosterone, growth hormone, and exercise, making it a potential early marker of muscle quality and anabolic response, particularly in men [85,86]. These findings underscore the importance of considering hormonal changes and sex differences when interpreting biomarker concentrations and their relationship to muscle health.

In summary, P3NP shows promise as a biomarker of muscle remodeling and anabolic activity. Although its response to short-term resistance training is modest, baseline P3NP levels may help predict lean mass gains [83]. Observational studies also suggest a sex-specific association between P3NP and muscle mass in men, though its utility as a diagnostic marker for sarcopenia appears limited [84]. These findings highlight the need to consider sex, hormones, and individual variability when evaluating P3NP as a marker of muscle health.

## 5. Limitations and Future Directions

Muscle myokines have emerged as potential biomarkers for muscle health, but their clinical application faces notable challenges. Some myokines are not muscle-specific and can be influenced by systemic inflammation, making it difficult to attribute their levels solely to muscle status. Inter-individual variability—including age, sex, diet, and physical activity levels—further complicates interpretation; we acknowledge that this review does not comprehensively address nutritional factors that may influence muscle. Additionally, the lack of standardized assays, population-specific reference ranges, and assay reproducibility limits comparability across studies. Cost considerations, access to specialized testing, and the complexity of integrating biomarker interpretation into routine clinical workflows also remain significant challenges.

Currently, most of the research in this area has been cross-sectional or short-term studies; longitudinal and large population interventional studies are a minority. This limits our ability to determine predictive value and establish causal relationships. Future directions should focus on identifying muscle-specific myokines with increased diagnostic specificity, improving assay sensitivity, reproducibility, and cost-effectiveness. Establishing standardized protocols and reference ranges across diverse populations and clinical settings is also critical. Research exploring the use of digital health tools such as wearable devices or remote monitoring in combination with studies on how to combine biomarker measurements with current functional assessments (e.g., grip strength or gait speed) could provide valuable insights into how these approaches can be integrated into everyday clinical practice.

## 6. Conclusions

Maintaining muscle health during aging is essential for preserving physical performance, independence, and quality of life. As muscle mass, strength, and function naturally decline over time, the risks of mobility impairment, frailty, and chronic diseases increase significantly. Early detection of muscle deterioration through reliable biomarkers is therefore critical for timely intervention—before irreversible damage occurs. While this review highlights several promising biomarkers—including IGF-1, myostatin, IL-6, irisin, IL-15, and P3NP—that offer valuable insight into different aspects of muscle physiology, pathology, and metabolic regulation, translation into routine clinical care is not yet imminent. Table 1 summarizes these myokines.

Significant barriers such as assay cost, lack of standardization, variability in biomarker interpretation, and uncertainty on how these markers perform in heterogeneous aging populations, remain of critical importance. Thus, rather than replacing already established performance-based assessments, biomarkers should be perceived as complementary measures that can enhance early detection, personalize interventions, and track therapeutic responses. Ultimately, a combined approach that integrates validated biomarker panels with standardized functional tests and clinical assessments, while leveraging emerging technologies such as digital health tools, has the potential to improve early detection, diagnosis, and management of age-related muscle decline. A comprehensive biomarker-based approach may enable healthcare providers to identify individuals at risk of muscle deterioration before it manifests as functional impairment. Finally, deepening our understanding of the biological mechanisms that drive muscle aging will enhance our ability to optimize interventions, promote healthy aging, and support a better quality of life for older adults. Achieving this will require rigorous longitudinal studies, synchronous testing protocols, and assessing real-world implementation to ensure feasibility, accuracy, and real-life clinical value.

## Figures and Tables

**Table 1:** Summary of key myokines.

Myokine	Origin	Mechanistic Pathways	Clinical Relevance	Notes
IGF-1	Liver, skeletal muscle	Stimulates muscle cell proliferation, differentiation, and survival Activates satellite cells Activates PI3K/Akt/mTOR ^1^ (↑ protein synthesis, ↓ breakdown)	Enhances muscle growth/repair Low levels → sarcopenia, frailty Correlates with muscle mass and strength	Sex- and age-dependent; influenced by diet, activity, hormones
Myostatin	Skeletal muscle	Inhibits muscle cell proliferation/differentiation Suppresses satellite cells Promotes atrophy via SMAD/MAPK ^2^	Negative regulator of growth Elevated → atrophy, poor regeneration, marker of sarcopenia risk	Sex-specific effects
IL-6	Skeletal muscle	Modulates inflammation and muscle metabolism Promotes repair via NF-κB ^3^, JAK/STAT ^4^ Activates satellite cells	Acute ↑ = beneficial (repair) Chronic ↑ = sarcopenia, cachexia	Context-dependent; chronic effects worse in aging women
Irisin	Skeletal muscle	Induces adipose browning ↑ Mitochondrial biogenesis Supports muscle metabolism and regeneration	Protects against atrophy Correlates with strength, endurance Linked to fat mass ↓	Training-responsive
IL-15	Skeletal muscle	Stimulates proliferation and regeneration Enhances oxidative capacity Regulates mitochondrial biogenesis	Supports lean mass and function Low levels → sarcopenia, poor outcomes	Sex-specific effects; involved in both muscle and fat regulation
P3NP	Skeletal muscle (collagen cleavage product)	Marker of collagen synthesis and muscle remodeling Reflects anabolic activity	Baseline levels predict lean mass gain Serves as an early marker of anabolic activity	Sex and hormone-dependent

Legend: ^1^ PI3K/Akt/mTOR = Phosphoinositide 3-kinase/Protein kinase B/Mechanistic target of rapamycin pathway; ^2^ SMAD/MPAK = Suppressor of Mothers Against Decapentaplegic/Mitogen-Activated Protein Kinase; ^3^ NF-κB = Nuclear Factor kappa-light-chain-enhancer of activated B cells; ^4^ JAK/STAT = Janus Kinase/Signal Transducer and Activator of Transcription. “↑” = increase; “↓” = decrease.
